# Early Exposure to Volatile Anesthetics Impairs Long-Term Associative Learning and Recognition Memory

**DOI:** 10.1371/journal.pone.0105340

**Published:** 2014-08-28

**Authors:** Bradley H. Lee, John Thomas Chan, Obhi Hazarika, Laszlo Vutskits, Jeffrey W. Sall

**Affiliations:** 1 Department of Anesthesia and Perioperative Care, University of California San Francisco, San Francisco, California, Unites States of America; 2 Department of Anesthesiology, Pharmacology and Intensive Care, University Hospital of Geneva, Geneva, Switzerland; Universidade do Estado do Rio de Janeiro, Brazil

## Abstract

**Background:**

Anesthetic exposure early in life affects neural development and long-term cognitive function, but our understanding of the types of memory that are altered is incomplete. Specific cognitive tests in rodents that isolate different memory processes provide a useful approach for gaining insight into this issue.

**Methods:**

Postnatal day 7 (P7) rats were exposed to either desflurane or isoflurane at 1 Minimum Alveolar Concentration for 4 h. Acute neuronal death was assessed 12 h later in the thalamus, CA1-3 regions of hippocampus, and dentate gyrus. In separate behavioral experiments, beginning at P48, subjects were evaluated in a series of object recognition tests relying on associative learning, as well as social recognition.

**Results:**

Exposure to either anesthetic led to a significant increase in neuroapoptosis in each brain region. The extent of neuronal death did not differ between groups. Subjects were unaffected in simple tasks of novel object and object-location recognition. However, anesthetized animals from both groups were impaired in allocentric object-location memory and a more complex task requiring subjects to associate an object with its location and contextual setting. Isoflurane exposure led to additional impairment in object-context association and social memory.

**Conclusion:**

Isoflurane and desflurane exposure during development result in deficits in tasks relying on associative learning and recognition memory. Isoflurane may potentially cause worse impairment than desflurane.

## Introduction

Every day, anesthetics are used around the world in newborns and infants who undergo medical procedures. There is growing concern that anesthetics can significantly alter the developing brain, and animal models have shown that exposure to anesthetics at an early age lead to neuronal death and long-term cognitive dysfunction [1]–[3]. Epidemiologic studies suggest that humans are also susceptible to long-term cognitive effects after anesthesia [4], [5].

Our knowledge of cognitive effects in humans has been, until recently [6], limited to retrospective studies that typically assess global tests of learning and behavior [4], [5], [7], [8]. For instance, most of these epidemiologic studies identify cognitive or learning disabilities by evaluating databases for individuals with diagnostic codes for unspecified delays, behavioral disorders, language or speech problems [7], [8], or through IQ and achievement tests [4], [5]. Because these studies examine generalized learning problems, they contribute minimally to our understanding of the memory processes that underlie the cognitive impairment.

An important challenge in the study of anesthetic neurotoxicity is developing a model by which cognitive effects in animals can be translated to humans. Memory processing is highly conserved across rodent and human species [9]. In particular, hippocampal memory functions are very similar between rats and humans [9], and the hippocampus is crucial in spatial encoding, associative learning, and recognition memory in both rats and humans [9]–[12].

Rodent models therefore provide valuable insight into the types of memory that may be affected in humans. However, behavioral studies are prone to using overlapping models for evaluating learning and memory. Many studies use similar tests, such as the Morris water maze [2], [13]–[15], because they have consistently identified a cognitive deficit. Identifying impairment in specific memory processes, such as recognition and associative memory, in animal models will provide insight into effects in humans and may help guide future assessments of learning and memory in children, as has recently been reported [6].

Recognition memory, which is a subtype of declarative memory, is crucial in humans for recalling different events, objects, and people [16], [17]. It has been shown that animals also have episodic-like memory that can be demonstrated through tests involving memory for “what,” “where,” and “when” details of an event. This was first described in birds [18] and more recently in rodents [12], [19]–[22], and models have since been developed to examine recognition memory in various ways [20], [23]–[25]. Furthermore, many studies find that recognition memory processes rely on the hippocampus and thalamus [19], [26], which are areas of neuronal degeneration following anesthesia [2], [14].

The present study was designed to evaluate the effects of two commonly used volatile anesthetics – isoflurane and desflurane – on specific learning and memory processes following neonatal exposure. After delivering 1 Minimum Alveolar Concentration [27] of either anesthetic for 4 hours at postnatal day 7 (P7), subjects were evaluated in a set of recognition tasks involving associative memory, as well as social memory, that have been shown to be sensitive to lesions in hippocampal and thalamic circuits [19], [28], [29].

## Methods

### Subjects

All experiments were conducted with approval from the Institutional Animal Care and Use Committee at the University of California, San Francisco. Five Sprague-Dawley dams with litters of postnatal day 6 (P6) pups from were obtained from Charles River Laboratories (Gilroy, CA). Each litter contained only males and was culled to ten pups. In total, the males were taken from at least ten different litters. On P7, animals from each litter were randomly assigned to control and treatment groups. They were weaned at P23 and housed three per cage under standard lab housing with 12 h light/dark cycle. Animals were food restricted (access to food only during light cycle) for tasks involving object recognition to increase activity and object exploration.

### Anesthesia

**Figure 1:**
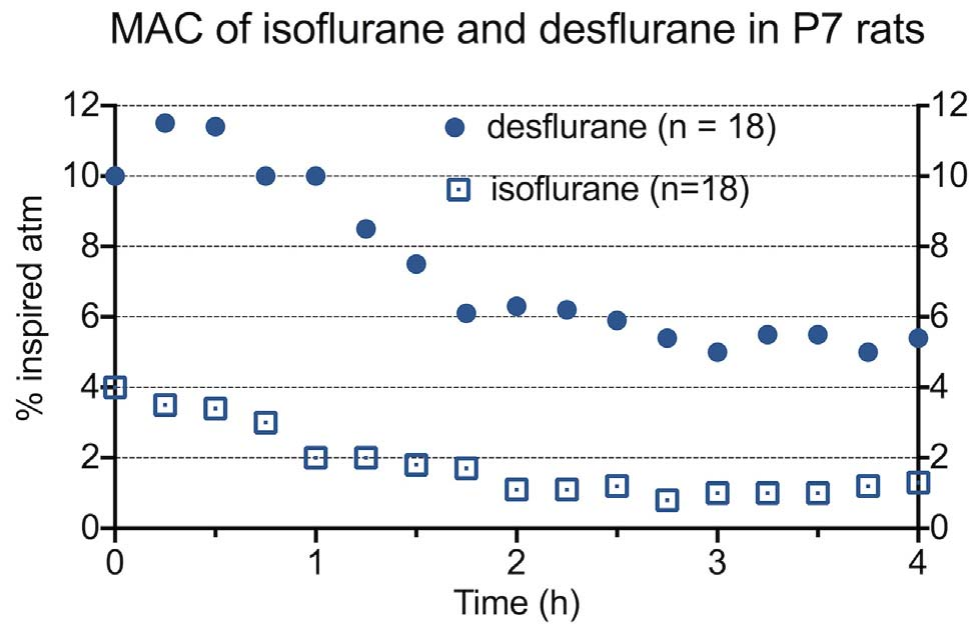
MAC of isoflurane and desflurane. Anesthetics were separately delivered to P7 rats in air and oxygen (FiO_2_ 50%) as previously described^6, 9^. Tail-clamping occurred every 15 minutes, and anesthetic concentration was adjusted to 1 MAC. As before^6, 9^, MAC decreases with increasing duration of anesthesia for both agents.

Anesthesia was delivered as described previously [14], [30], [31]. Briefly, animals in the treatment groups received either isoflurane or desflurane as a single agent in air and oxygen (FiO_2_50%) at 1 Minimum Alveolar Concentration [27] for four hours. MAC was determined by tail clamping every 15 minutes, and anesthetic concentration was adjusted accordingly, so that on average 50% of animals would move in response to clamping (Fig. 1). 12 out of 18 animals anesthetized with isoflurane survived to undergo behavioral testing, and 13 out of 18 animals anesthetized with desflurane survived and underwent behavioral testing. Control animals were concurrently placed in an anesthesia glove box of the same material and conditions without being exposed to anesthesia or tail clamping. Animals were kept on a warming blanket, and temperatures were measured using an infrared laser thermometer and maintained with a goal of 35°C.

### Histology

Brains from the two anesthetized groups and the control group (n = 10 per group) were assessed for acute neuronal death. Twelve hours after anesthesia, animals were transcardially perfused with cold 4% paraformaldehyde in phosphate-buffered saline and brains were removed, postfixed, and sunk in sucrose solution. They were then sliced into 60 micron-thick slices and every other slice was mounted and stained with FluoroJade C, a marker specific for neurodegeneration [32], [33] (FJC, 0.001%, Millipore, Billerica, MA). FJ-positive cells were counted using Nikon Eclipse *80i* microscope under 20X magnification in each slice containing the structure of interest. Structures included in analysis were the anterodorsal (AD), anteroventral (AV), laterodorsal (LD), and anteromedial (AM) thalamic nuclei, as well as CA1-3 regions of the hippocampus and the dentate gyrus.

### Object Recognition Tasks

Object recognition was assessed using similar arrangements as others [19], [28]. Behavior testing occurred during the light phase of the circadian cycle between 0800 and 1700 hrs in two separate arenas, hereafter referred to as contexts, of identical size (61 cm square base, walls 50 cm high). Context 1 had yellow walls with a base covered in wood-effect vinyl lining, and context 2 had black walls with a black plastic base. Different visual cues were placed on the walls of each context. A video camera (SONY HDR-CX190) was mounted 2 meters above the testing area for recording and observing subjects. For each task, except the allocentric object-location task, subjects were placed into contexts in the same location and facing the south wall (away from the objects). Beginning at P42, subjects were habituated to the two contexts prior to testing by being placed individually into the context for 5 min per day for 4 consecutive days. All animals underwent all behavioral tasks. Subjects were tested on the same day for any given task and in the same sequence of tasks. All tasks were performed in the order presented in subsequent weeks, except for the first two (novel object and object-place) which were performed in the same week. The order of testing during the day was counterbalanced among groups.

Investigation of an object was defined as sniffing or placing the nose within 1 cm of and oriented toward the object. Subjects were recorded, and observers blinded to group assignment were used to determine investigation times. Object investigation times during the initial exposure for each task were compared to assess for possible confounding effects of varying investigation times on the ability to recognize objects. All objects and testing arenas were wiped with 70% ethanol between testing.

#### Novel Object Recognition

**Figure 2:**
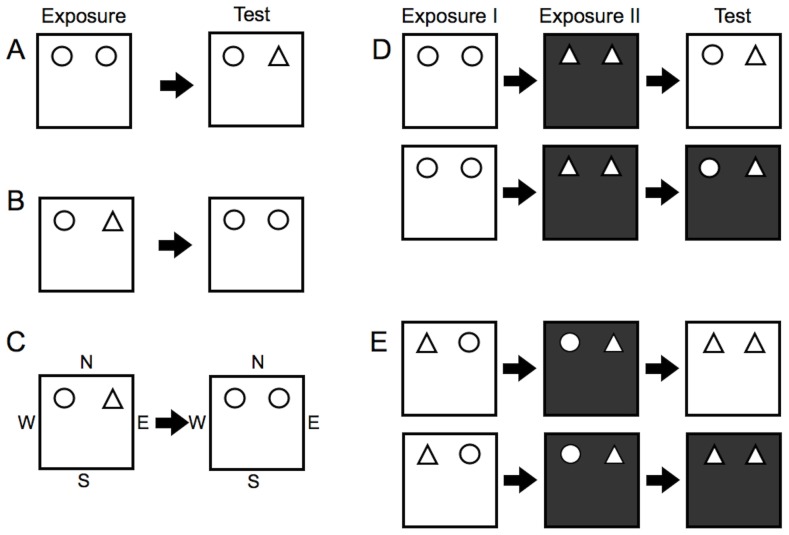
Object recognition. For each task, except allocentric object-place recognition, subjects are introduced at and facing the wall away from the objects. (A) *Novel object recognition.* Two identical objects are presented in the exposure, and one (right) is replaced with a novel object in the test phase. (B) *Object-place recognition*. Two different objects are presented, followed by two identical objects. In the test phase, the right object appears in a novel location within the context. (C) In the allocentric version of object-place recognition, subjects are again introduced at and facing the south wall (S) in the exposure. However, for the test phase, subjects are placed at and facing either the east (E) or west (W) wall. (D) *Object-context recognition*. Two different pairs of objects are presented in two different contexts, so each object is associated with a particular context. In the test phase, one object (right object, top row; left object, bottom row) appears within a context in which it has not been explored. (E) *Object-place-context recognition*. Two different objects are first presented in a context. The object locations are then reversed and presented in a different context. Thus, after two exposures, each object is seen in both contexts and both locations (left and right). In the test phase, two objects are presented in either context, so one (right object, top row; left object, bottom row) appears in a novel configuration of place and context.

Testing began at P48 with novel object recognition. A single trial was performed for each animal consisting of “exposure” and “test” phases separated by a two-minute delay (Fig. 2A). During the exposure, subjects were placed into the context and allowed to explore two *identical* objects for four minutes. After the delay, they were placed into the same context for three minutes with one of the objects replaced with a novel object. Half of the subjects were tested in each context with the location (left or right) of the novel object counterbalanced among subjects.

#### Object-Place Recognition

Subjects were tested in their ability to recognize an object and its location. Two trials were performed, and investigation times were totaled for the two trials. In the exposure, two *different* objects were presented in a context for four minutes. After a two-minute delay, two *identical* copies of one of the previous objects were presented in the same context for three minutes (Fig. 2B). Both objects were equally familiar, but one now occupied a different location within the context.

#### Allocentric Object-Place Recognition

For the previous task, subjects were always introduced into the context facing the wall (south wall) opposite the two objects (Fig. 2C). In the allocentric version of the task, for the initial exposure, subjects were again placed into the context facing the south wall. In the test phase, however, the entry point was varied and half of the subjects were introduced facing either the east or west wall (Fig. 2C). Two trials were performed and the entry point was randomized among subjects.

#### Object-Context Recognition

Subjects were assessed in their ability to recognize an object with a particular context. The task required two separate exposures, each lasting four minutes and separated by a two-minute delay (Fig. 2D). In the first exposure, a pair of *identical* objects was presented in a context. Next, subjects were placed in a *different* context with a *different* pair of objects. In the test phase, lasting three minutes, subjects were placed into a context with one of each previously encountered object. Thus, one object was presented in the same context as before, while the other object appeared within a context in which it had not been explored. Two trials were conducted, and the test phase occurred in opposite contexts for each trial (Fig. 2D).

#### Object-Place-Context Recognition

Subjects were tested in their ability to recognize an object with its location and context (Fig. 2E). In the first exposure, two *different* objects were presented within a context. Next, subjects were placed in the opposite context with the *same* two objects and their locations reversed. Thus, after two exposures, each object was observed in both contexts and locations (left and right). In the test phase, two *identical* copies of either of the previous objects were presented in a context. The location and context associated with one object were familiar, while the other “displaced” object appeared in a location and context in which it had not been observed. Two trials were conducted with the test phase occurring in opposite contexts for each trial (Fig. 2E).

### Social Behavior and Social Recognition

Following object recognition, animals were given unrestricted access to food. Social interaction and recognition were assessed using a discrimination paradigm one week after completing object recognition testing at P80. In the exposure, the subject was presented with a caged stimulus animal and a novel object for five minutes. This arrangement evaluates social behavior by determining whether subjects spend more time investigating the stimulus animal or object^7^. After a sixty-minute delay, subjects were presented simultaneously with the same “familiar” animal and a novel animal for three minutes. Recognition of the previously encountered animal was demonstrated by decreased investigation of the familiar target relative to the novel one.

Same-sex juvenile conspecifics were used as stimulus animals. Male pups five weeks of age were housed individually one week prior to testing. Investigation of the stimulus animal was defined as sniffing or direct contact with the subject’s nose or paws. Investigation of the novel object was defined as sniffing or placing the nose within 1 cm of and oriented toward object.

### Statistical Analysis

Data were analyzed using Prism 6 Software for Mac OSX (GraphPad Software Inc., San Diego, CA). Data were assessed for normal distribution using the D’Agostino and Pearson test. Parametric tests were used for normally distributed data; otherwise, nonparametric tests were used for analysis. All comparisons used a two-tail test and a P value less than 0.05 was considered statistically significant.

Total FluoroJade-positive cells for each brain region were compared among the groups – control, desflurane, isoflurane – using one-way ANOVA for parametric data or the Kruskal-Wallis test for nonparametric data. Bonferroni’s post-test with multiple comparisons was used following one-way ANOVA, and Dunn’s post-test was used with the Kruskal-Wallis test. The fold-increase in neuronal death was determined for each structure by dividing the total FJ-positive cells for all anesthetized animals (n = 20) by the average number of FJ-positive cells per structure for control animals (n = 10).

Recognition tasks were first assessed by comparing the investigation times of each target using paired tests for each group. Paired t-test was used for normally distributed data, and nonparametric data were analyzed with the Wilcoxon matched-pairs rank test. Also, to identify possible confounding effects of varying investigation times on subsequent object/animal recognition, the times during the exposure phase were compared between the groups using either one-way ANOVA with Bonferroni’s post-test or the Kruskal-Wallis test with Dunn’s post-test.

In addition, a “discrimination index” (DI) was calculated and represents the relative time spent exploring each target (eg. Familiar versus Novel). To calculate DI, the time spent investigating the familiar target was subtracted from the time spent on the novel target, and this was divided by the total time spent investigating the two (eg. DI = (Novel-Familiar)/(Total Time)). This value was compared to a theoretical value of zero using one sample t-test to assess whether a preference was shown for one of the objects, and a positive DI indicates preference for the novel aspect of the task. For each task, DI of control animals was compared against DI of all anesthetized animals. Also, within the group of anesthetized animals, the DI of desflurane-treated subjects was compared with that of isoflurane-treated subjects. These comparisons were made using either unpaired t-test for parametric data or the Mann Whitney test for nonparametric data.

## Results

### Increased neuronal death occurs similarly in desflurane and isoflurane-treated animals

**Figure 3:**
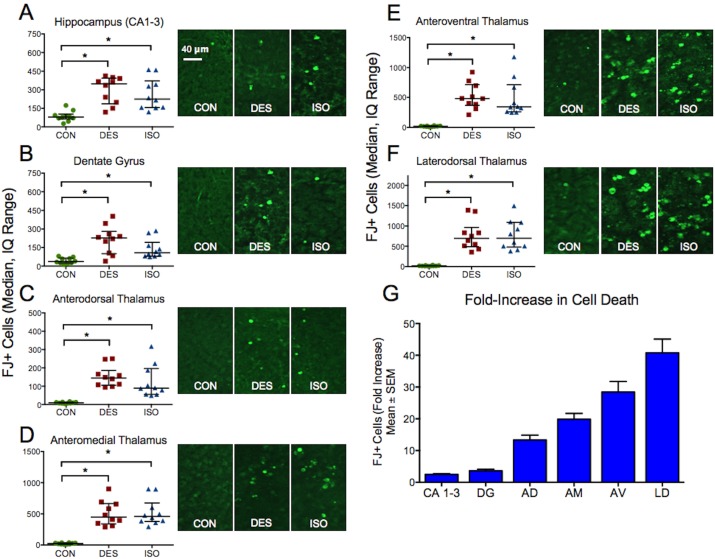
Neuronal death by group. **A** to **F)** Exposure to either anesthetic – desflurane or isoflurane – led to significantly increased neuronal death in each brain region. The degree of neurodegeneration was similar in desflurane and isoflurane-treated subjects. Sample images from brains at 20X magnification are shown alongside graphs comparing total cell death for each structure. **G)** The average increases in neuronal death relative to controls are shown. *P<0.05.

There was increased neuronal death in each brain region in animals exposed to either desflurane or isoflurane relative to the control animals (Fig. 3). No difference in the extent of cell death was identified between the two anesthetized groups. Anesthetic exposure resulted in significantly increased cell death in the hippocampus (P = 0.0001, one-way ANOVA; control vs. des P = 0.0002, control vs. iso P = 0.0015, des vs. iso P = 0.99, Bonferroni), dentate gyrus (P = 0.0003, one-way ANOVA; control vs. des P = 0.0002, control vs. iso P = 0.03, des vs. iso P = 0.16, Bonferroni), anterodorsal thalamus (P<0.0001, one-way ANOVA; control vs. des P<0.0001, control vs. iso P = 0.0007, des vs. iso P = 0.98, Bonferroni), anteromedial thalamus (P<0.0001, one-way ANOVA; control vs. des P<0.0001, control vs. iso P<0.0001, des vs. iso P = 0.99, Bonferroni), anteroventral thalamus (P<0.0001, Kruskal-Walli test; control vs. des P<0.0001, control vs. iso P = 0.001, des vs. iso P = 0.99, Dunn’s), and laterodorsal thalamus (P<0.0001, one-way ANOVA; control vs. des P<0.0001, control vs. iso P<0.0001, des vs. iso P = 0.99, Bonferroni). The relative fold-increase in cell death for each brain region is shown in Figure 3G.

### Novel Object and Object-Place Recognition are Unaffected

**Figure 4:**
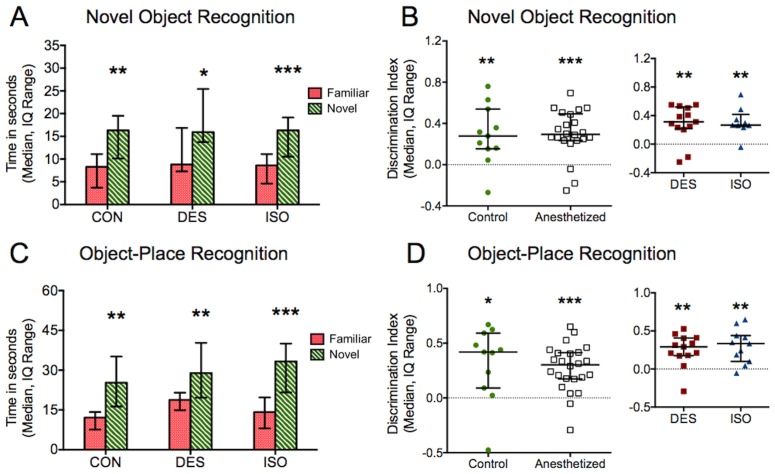
Novel object and object-place recognition. A) Subjects all demonstrated successful object recognition and preferentially explored the novel object. B) Each group’s DI was significantly greater than zero, and there was no difference in DIs. C) Subjects were also able to identify an object in a novel location, demonstrated by a relative increase in investigation of that object. D) Again, DIs for all subjects were greater than zero with no differences identified. *P<0.05, **P<0.01, ***P<0.001, CON = control, DES = desflurane, ISO = isoflurane.

Subjects from each group were able to distinguish familiar and novel objects, revealed by increased investigation times of the novel object (control P = 0.006, desflurane P = 0.01, isoflurane P = 0.0003; paired t-test familiar vs. novel, Fig. 4A). Object-place recognition was also intact in each group, and animals spent more time with the object in a novel location (control P = 0.006, desflurane P = 0.001, isoflurane P = 0.0008, paired t-test familiar vs. novel location, Fig. 4C). There was no difference in object exploration times among groups during the exposure for either task (novel object P = 0.5, one-way ANOVA, object-place P = 0.2, Kruskal-Wallis).

Discrimination Indexes [3] for all subjects were greater than zero for both novel object recognition (control P = 0.007, desflurane P = 0.002, isoflurane P = 0.002, one sample t-test, Fig. 4B) and object-place recognition (control P = 0.01, desflurane P = 0.001, isoflurane P = 0.001, one sample t-test, Fig. 4D). No differences in DI were identified between control and anesthetized subjects (novel object P = 0.9, unpaired t-test; object-place P = 0.3, Mann Whitney test) or between desflurane and isoflurane subjects (novel object P = 0.83, unpaired t-test; object-place P = 0.64, Mann Whitney test).

### Isoflurane but not desflurane treated animals are impaired in object-context and social recognition

**Figure 5:**
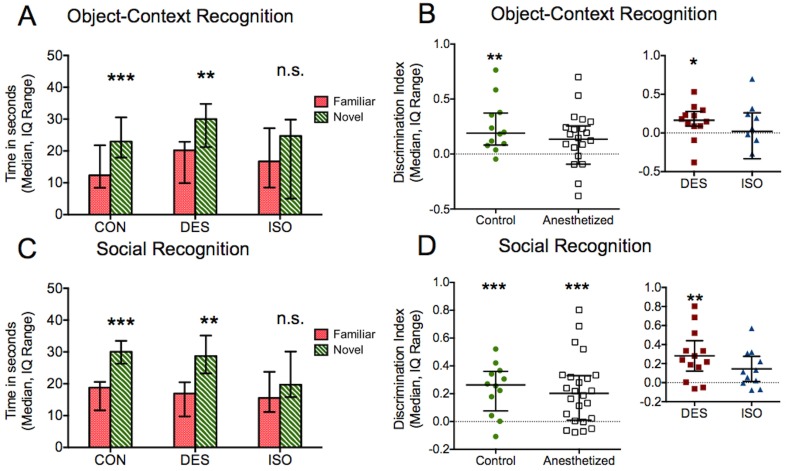
Object-context and social recognition. A) Isoflurane-treated animals were impaired in associating an object with a particular context. Animals exposed to desflurane, on the other hand, recognized when an object appeared in a different context and spent more time with that object. B) The DI for anesthetized subjects in this task did not differ from zero, and, within this group, only the desflurane DI significantly exceeded zero. C) Desflurane-treated subjects also had no change in social recognition ability, spending more time with the novel animal, while isoflurane-treated animals had deficient social memory. D) DI for both control and anesthetized animals exceeded zero, although DI for the subset of isoflurane-treated subjects did not. *P<0.05, **P<0.01, ***P<0.001, n.s. = not significant.

Only the isoflurane group was impaired in the ability to associate an object with its context and spent similar amounts of time with each object in this task (control P = 0.001, Wilcoxon test familiar vs. novel context; desflurane P = 0.006, isoflurane P = 0.2, paired t-test, Fig. 5A). DIs of control and desflurane subjects were greater than zero but not in isoflurane-treated subjects (control P = 0.004, desflurane P = 0.04, isoflurane P = 0.95, one sample t-test, Fig. 5B). Comparison of DI between control and anesthetized subjects did not reveal a difference (P = 0.094, unpaired t-test, Fig. 5B). Within the anesthetized group, DI did not differ significantly between desflurane and isoflurane-treated subjects (P = 0.32, unpaired t-test). Exploration times in the exposure phases of the object-context task were similar for the three groups (P = 0.6, one-way ANOVA).

Isoflurane animals also had impaired social memory while desflurane animals were unaffected when comparing social target investigation times (control P = 0.0009, desflurane P = 0.002, isoflurane P = 0.08; paired t-test familiar vs. novel animal, Fig. 5C). DIs of control and desflurane subjects were greater than zero (control P = 0.0009, desflurane P = 0.002, one sample t-test, Fig. 5D), although isoflurane DI did not differ significantly from zero (P = 0.064, one sample t-test, Fig. 5D). No difference between DI was identified in control vs. anesthetized groups (P = 0.84, unpaired t-test). In the subset of anesthetized subjects, the isoflurane DI was lower than desflurane DI although it did not reach statistical significance (P = 0.17, unpaired t-test). In the exposure of the social recognition task, animals from all groups displayed normal social behavior and spent significantly greater time investigating the social target relative to the object (all P<0.0001, paired t-test object vs. social target).

### Anesthetized subjects are impaired in allocentric object-place and object-place-context recognition

**Figure 6:**
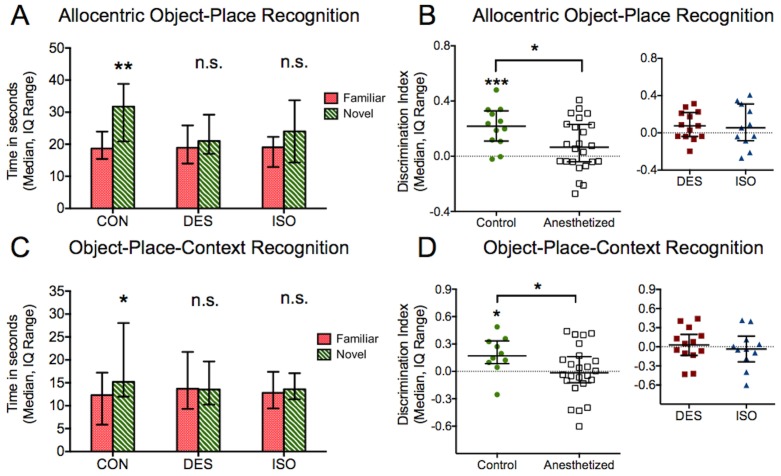
Allocentric object-place and object-place-context recognition. A) Exposure to isoflurane or desflurane led to impairment in identifying an object’s location when the site of entry into the context was changed. The varied entry points forced subjects to rely on allocentric cues to identify the object’s location. B) DI of control animals was significantly greater than that of anesthetized subjects. Neither desflurane nor isoflurane DI significantly exceeded zero. C) Isoflurane and desflurane-treated subjects were also impaired in recognition of an object that required association of its place and context. D) Again, control DI was greater than anesthetized DI. Neither subset of anesthetized subjects – desflurane or isoflurane – had DI greater than zero. *P<0.05, **P<0.01, n.s. = not significant.

Animals from both isoflurane and desflurane groups were impaired in object recognition when the entry site was varied in the allocentric version of the object-place task (control P = 0.001, desflurane P = 0.08, paired t-test familiar vs. novel; isoflurane P = 0.2, Wilcoxon test, Fig. 6A). The control DI was greater than zero (P = 0.0004, one sample t-test, Fig. 6B), while neither desflurane nor isoflurane DI differed from zero (desflurane P = 0.094, isoflurane P = 0.31, one sample t-test, Fig. 6B). DI of control animals was also significantly greater than that of anesthetized subjects (P = 0.024, unpaired t-test), although no difference was detected in the subset of desflurane and isoflurane-treated animals (P = 0.95, unpaired t-test).

Anesthetized subjects from both groups were also unable to distinguish objects in the object-place-context task (control P = 0.04, desflurane P = 0.5, paired t-test familiar vs. displaced; isoflurane P = 0.8, Wilcoxon test, Fig. 6C). Only the control DI exceeded zero in this task (control P = 0.021, desflurane P = 0.71, isoflurane P = 0.7, one sample t-test, Fig. 6D). Control DI was again significantly greater than DI for anesthetized subjects (P = 0.04, unpaired t-test), and no difference was found between desflurane and isoflurane DIs (P = 0.59, unpaired t-test). Investigation times during the exposures were similar between groups for each task (allocentric object-place P = 0.1, object-place-context P = 0.7, one-way ANOVA). The summary of all behavioral testing is presented in Table 1, where each group is evaluated whether they demonstrate a preference for the novel portion of the task by recognizing a familiar set of stimuli.

**Table 1:** Summary of behavioral testing.

	Discrimination Index for task greater than zero?		
	Control	DES	ISO
Novel Object Recognition	Yes	Yes	Yes
Object-Place Recognition	Yes	Yes	Yes
Object-Context Recognition	Yes	Yes	***No***
Social Recognition	Yes	Yes	***No***
Allocentric Object-Place Recognition	Yes	***No***	***No***
Object-Place-Context Recognition	Yes	***No***	***No***

For each test, recognition of a familiar set of stimuli results in preferential exploration of the novel aspect of the task. Discrimination Index (DI) represents the time spent with the novel object or animal relative to the familiar one, and DI significantly greater than zero demonstrates successful recognition in the task.

## Discussion

The main finding of this study is that exposure to the volatile anesthetics isoflurane and desflurane causes impairment in tasks relying on specific cognitive processes of associative learning and recognition memory. After exposure to 1 MAC of either anesthetic for 4 hours during the early postnatal period, adult subjects could identify a novel object and recognize changes in an object’s spatial location. However, anesthetized animals were unable to recognize an object’s location when they entered the testing arena from a different vantage point or perform a complex task requiring the integration of object, place, and context details. In addition, isoflurane-treated subjects were impaired in context-specific object recognition and exhibited deficient social memory.

The behaviors assessed in this study provide valuable insight into the types of learning affected by neonatal anesthesia exposure. The object recognition tasks performed here rely on spatial memory, but they also require associative processing to encode the relationships among distinct elements encountered during a given exposure [28], [34], [35]. Both control and treatment animals easily recognize a novel object, but animals that were anesthetized on P7 begin to show impairment when presented with objects that were previously in a different location or context, suggesting problems with associative learning. The impairment in the allocentric object-place task may also be related to spatial memory, because the animals are able to identify objects when relying on egocentric cues but struggle when forced to rely on allocentric cues.

Episodic memory is associative in nature, and memory formation relies in large part on our ability to link new experiences and items with closely related ideas, facts, and the environment or context in which we learn them [36]. Clearly, a problem forming associations and relationships would affect memory encoding over time. Furthermore, within the broad domain of episodic memory, recognition memory is a specific type of memory that, according to the dual process model, is comprised of recollection and familiarity [26], [36]. It is likely that impairment in the object recognition and associative memory tasks could also result from a deficit in recollection, a process underlying recognition memory [19], [28]. We recently reported deficits in recollection in both rodents and children after anesthesia at an early age [6]. Persistent problems with associative and recognition memory in children would have important consequences for learning and development throughout adolescence. The precise cognitive domains that may be impaired in children and how these effects manifest later in life is still unclear, and these are important areas of future investigation.

Isoflurane has been used in numerous studies to investigate the effects of anesthesia and many labs have reported cell death and behavioral changes after isoflurane exposure [1]–[3], [14], [30]. The effects associated with desflurane, though, are less well described. Similar to other volatile anesthetics, desflurane in neonates has been shown to induce cell death [37], [38]. However, few studies of behavior have been performed, and only one of these has demonstrated cognitive impairment [38]. Kodama and colleagues found that mice exposed to desflurane later developed problems with short-term and long-term memory [38]. In our present study, we demonstrate impairment in desflurane-treated animals using two separate tasks that involve associative learning. Together, these behavioral results show that desflurane, like isoflurane [2], [14], [30] and sevoflurane [13], [30], [39], alters long-term cognitive behavior.

Isoflurane-treated animals were impaired in two additional behavioral tasks, suggesting a distinct outcome from those anesthetized with desflurane. Others have also identified distinct outcomes using different anesthetic agents [37], [38], [40], [41], although the reason underlying these behavioral findings is unclear. The types of memory involved in this series of behavioral testing are processed in the medial temporal lobe [19], [28], including hippocampus and dentate gyrus, as well as the anterior thalamus and prefrontal cortex [26],[42], and we identified increased neurodegeneration in each of these brain regions. However, the observation of distinct behavioral outcomes occurred in the setting of a similar extent of neuronal injury. The discrepancy between histologic and behavioral findings suggest that, although neuronal death may play a role in determining behavioral phenotype, other effects on neural development likely contribute, as well. In fact, there is evidence that volatile anesthetics can alter synaptogenesis and dendritic spine density even in the absence of cell death [43]. In addition, anesthetics have been shown to result in significant neuroinflammation [41], changes in cell signaling [44], and stem cell proliferation [45], [46]. It is likely that anesthetic effects on these processes of brain development contribute to the ultimate cognitive outcome.

Isoflurane-treated animals also had difficulty with social recognition which is more likely related to long-term memory processes than their capacity for social interaction. Unlike previous reports [39], we found all animals behaved similarly during the exposure portion of the test, spending much more time with a novel animal than an object. In fact, throughout these experiments none of the treatment groups demonstrated a difference in exploration time during the exposure phase. This suggests anesthetic exposure does not alter investigatory or social behavior, motivation, or attention.

### Limitations

The purpose of this study is to evaluate two separate anesthetics using outcomes of cell death and behavior. We cannot make conclusive remarks regarding mechanisms underlying cognitive impairment, and separate studies are needed to better understand these processes. Also, a comprehensive analysis of neuronal death was not undertaken, and it is possible that other brain regions show a difference. The hippocampus and thalamus were chosen, however, because of their underlying role in the investigated behavior.

Social recognition is based on olfaction in rodents [47] and we did not perform a separate experiment to exclude impaired olfaction as the basis for deficient social recognition in our subjects. However, we have previously determined that anesthetic exposure does not impair olfaction [6]. Isoflurane-treated subjects displayed typical social behavior in each part of the test, suggesting impaired recognition was due to effects on memory rather than interest, motivation, or olfaction. Also, although the rats were tested serially, they did not show any signs of decreasing interest with the objects as we used objects that appeared novel to the subjects in each trial. In fact, the exploration times remained very similar across the tests from first to last.

There are numerous studies documenting effects of gestational and early life stress on long-term behavior [48], [49]. Because the animals were shipped, rather than bred in the housing facility, it is possible that they were exposed to early life stress that may affect aspects of behavior. Although effects of stress are likely evenly distributed amongst behavior groups, these considerations should be taken into account when interpreting behavioral results.

Finally, the cognitive outcomes from the two anesthetics appear to be different; however, it is possible that the two anesthetics were not entirely equal in depth in spite of being adjusted to 1 MAC. This must be taken into consideration when attempting to make direct comparisons between the two volatile anesthetics.

### Conclusion

Neonatal exposure to isoflurane and desflurane led to impairment in object recognition tasks relying on spatial and associative memory. These findings provide evidence that anesthetics can affect distinct cognitive processes that are fundamental to learning and memory in rodents, as well as humans.
